# Darwin's neuroscientist: Gerald M. Edelman, 1929–2014

**DOI:** 10.3389/fpsyg.2014.00896

**Published:** 2014-08-14

**Authors:** Anil K. Seth

**Affiliations:** Department of Informatics, Sackler Centre for Consciousness Science, School of Informatics and Engineering, University of SussexBrighton, UK

**Keywords:** consciousness, neural Darwinism, gamma globulin, reentry, selection

“The brain is wider than the sky.

For, put them side by side,

The one the other will include,

With ease, and you beside.”

Dr. Gerald M. Edelman often used these lines from Emily Dickinson to introduce the deep mysteries of neuroscience and consciousness. Dr. Edelman (it was always “Dr.”), who has died in La Jolla, aged 84, was without doubt a scientific great. He was a Nobel laureate at the age of 43, a pioneer in immunology, embryology, molecular biology, and neuroscience, a shrewd political operator, and a Renaissance man of striking erudition who displayed a masterful knowledge of science, music, literature, and the visual arts who at one time could have been a concert violinist. He quoted Woody Allen and Jascha Heifetz as readily as Linus Pauling and Ludwig Wittgenstein, a compelling raconteur who loved telling a good Jewish joke just as much as explaining the principles of neuronal selection. And he was my mentor from the time I arrived as a freshly minted Ph.D. at the Neuroscience Institute in San Diego, in 2001. His influence in biology and the neurosciences is inestimable. While his loss marks the end of an era, his legacy is sure to continue.

Gerald Maurice Edelman was born in Ozone Park, New York City, in 1929, to parents Edward and Anna (see Figure 1A). He trained in medicine at the University of Pennsylvania, graduating *cum laude* in 1954. After an internship at the Massachusetts General Hospital and three years in the US Army Medical Corp in France, Edelman entered the doctoral program at Rockefeller University, New York. Staying at Rockefeller after his Ph.D. he became Associate Dean and Vincent Astor Distinguished Professor, and in 1981 he founded The Neurosciences Institute (NSI). In 1992 the NSI moved lock, stock, and barrel into new purpose-built laboratories in La Jolla, California, where Edelman continued as Director for more than twenty years. A dedicated man, he continued working at the NSI until a week before he died.

**Figure 1:**
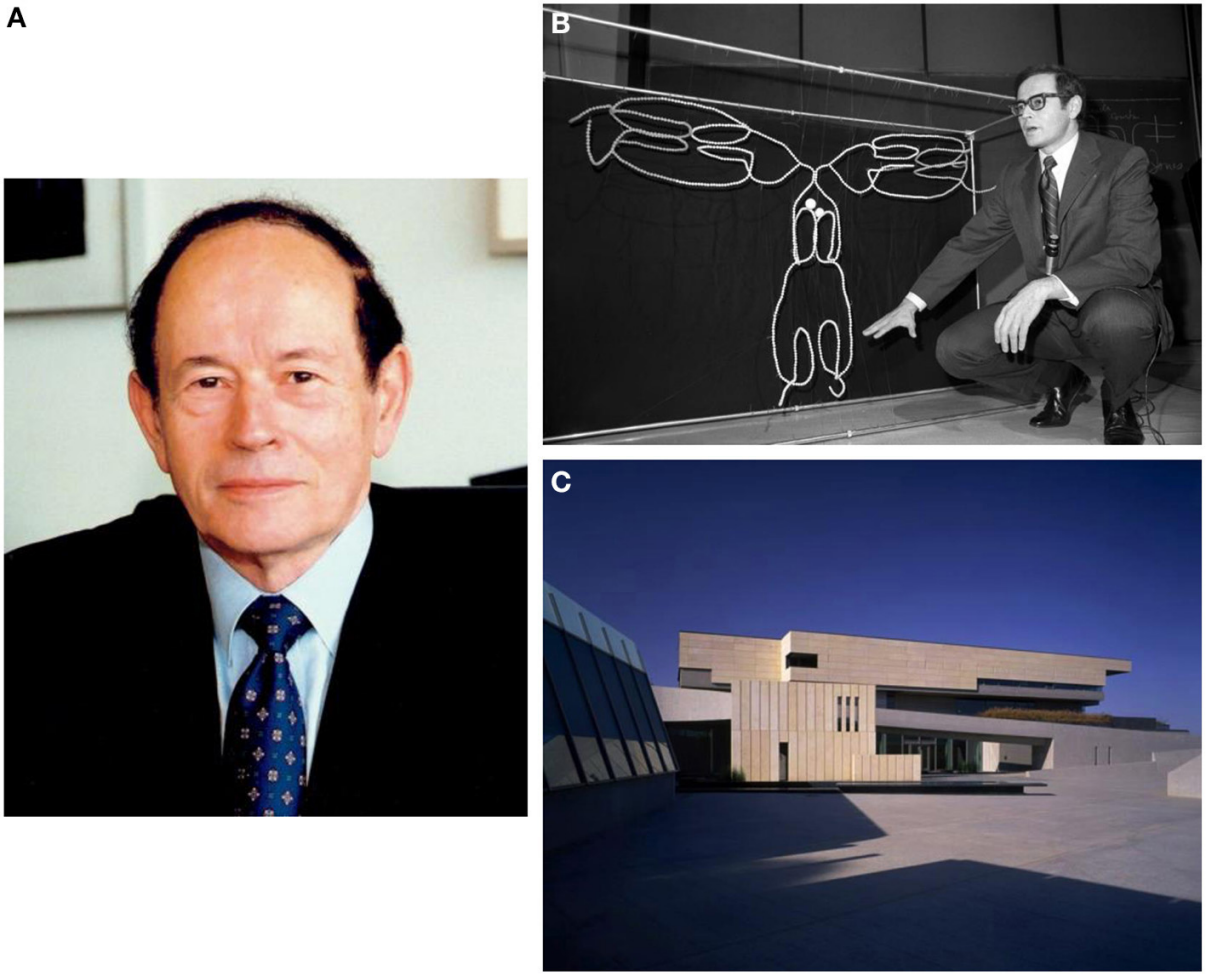
**(A)** Gerald M. Edelman, 1929–2014. **(B)** Dr. Edelman at Rockefeller University in 1972, explaining his model of gamma globulin. **(C)** The campus of The Neurosciences Institute in La Jolla, California.

In 1972 Edelman won the Nobel Prize in Physiology or Medicine (shared independently with Rodney Porter) for showing how antibodies can recognize an almost infinite range of invading antigens. Edelman's insight, the principles of which resonate throughout his entire career, was based in *variation* and *selection*: antibodies undergo a process of “evolution within the body” in order to match novel antigens. Crucially, he performed definitive experiments on the chemical structure of antibodies to support his idea (Edelman et al., 1961) (Figure 1B).

Edelman then moved into embryology, discovering an important class of proteins known as “cell adhesion molecules” (Edelman, 1983). Though this, too, was a major contribution, it was the biological basis of mind and consciousness—one of the “dark areas” of science, where mystery reigned - that drew his attention for the rest of his long career. Over more than three decades Edelman developed his theory of neuronal group selection, also known as “neural Darwinism,” which again took principles of variation and selection, but here applied them to brain development and dynamics (Edelman, 1978, 1987, 1989, 1993; Edelman and Gally, 2001). The theory is rich and still underappreciated. At its heart is the realization that the brain is very different from a computer: as he put it, brains don't work with “logic and a clock.” Instead, Edelman emphasized the rampantly “re-entrant” connectivity of the brain, with massively parallel bidirectional connections linking most brain regions. Uncovering the implications of re-entry remains a profound challenge today.

Edelman was convinced that scientific breakthroughs require both sharp minds and inspiring environments. The NSI was founded as a monastery of science, supporting a small cadre of experimental and theoretical neuroscientists and enabling them to work on ambitious goals free from the immediate pressures of research funding and paper publication (Figure 1C). This at least was the model, and Edelman struggled heroically to maintain its reality in the face of increasing financial pressures and the shifting landscape of academia. That he was able to succeed for so long attests to his political nous and focal determination as well as his intellectual abilities. I remember vividly the ritual lunches that exemplified life at the NSI. The entire scientific staff ate together at noon every day (except Fridays), at tables seemingly designed to hold just enough people so that the only common topic could be neuroscience; Edelman, of course, held court at one table, brainstorming and story-telling in equal measure. The NSI itself is a striking building, housing not only experimental laboratories but also a concert-grade auditorium. Science and art were, for Edelman, two manifestations of a fundamental urge toward creativity and beauty.

Edelman did not always take the easiest path through academic life. Among many rivalries, he enjoyed lively clashes with fellow Nobel laureate Francis Crick who, like Edelman himself, had turned his attention to the brain after resolving a central problem in a different area of biology. Crick once infamously referred to neural Darwinism as “*neural Edelmanism*” (Crick, 1989), a criticism which nowadays seems less forceful as attention within neurosciences increasingly focuses on neuronal population dynamics (just before his death in 2004, Crick met with Edelman and they put aside any remaining feelings of enmity). In 2003 both men published influential papers setting out their respective ideas on consciousness (Crick and Koch, 2003; Edelman, 2003); these papers put the neuroscience of consciousness at last, and for good, back on the agenda.

The biological basis of consciousness had been central to Edelman's scientific agenda from the late 1980s. Consciousness had long been considered beyond the reach of science; Edelman was at the forefront its rehabilitation as a serious subject within biology. His approach was from the outset more subtle and sophisticated than those of his contemporaries. Rather than simply looking for “*neural correlates of consciousness*”—brain areas or types of activity that happen to co-exist with conscious states—Edelman wanted to naturalize phenomenology itself. That is, he tried to establish formal mappings between phenomenological properties of conscious experience and homologous properties of neural dynamics. In short, this meant coming up with *explanations* rather than mere *correlations*, the idea being that such an approach would demystify the dualistic schism between “mind” and “matter” first invoked by Descartes. This approach was first outlined in his book *The Remembered Present* (Edelman, 1989) and later amplified in *A Universe of Consciousness*, a work co-authored with Giulio Tononi (Edelman and Tononi, 2000). It was this approach to consciousness that first drew me to the NSI and to Edelman, and I was not disappointed. These ideas, and the work they enabled, will continue to shape and define consciousness science for years to come.

My own memories of Edelman revolve entirely around life at the NSI. It was immediately obvious that he was not a distant boss who might leave his minions to get on with their research in isolation. He was generous with his time. I saw him almost every working day, and many discussions lasted long beyond their allotted duration. His dedication to detail sometimes took one's breath away. On one occasion, while working on a paper together, I had fallen into the habit of giving him a hard copy of my latest effort each Friday evening. One Monday morning I noticed the appearance of a thick sheaf of papers on my desk. Over the weekend Edelman had cut and paste—with scissors and glue, not Microsoft Word—paragraphs, sentences, and individual words, to almost entirely rewrite my tentative text. Needless to say, it was much improved.

The abiding memory of anyone who has spent time with Dr. Edelman is however not the scientific accomplishments, not the achievements encompassed by the NSI, but instead the impression of an uncommon intellect moving more quickly and ranging more widely than seemed possible. The *New York Times* put it this way in a 2004 profile (March 27, 2004):

“Out of free-floating riffs, vaudevillian jokes, recollections, citations and patient explanations, out of the excited explosions of example and counterexample, associations develop, mental terrain is reordered, and ever grander patterns emerge.”

Dr. Edelman will long be remembered for his remarkably diverse scientific contributions, his strength of character, erudition, integrity, and humor, and for the warmth and dedication he showed to those fortunate enough to share his vision. He is survived by his wife, Maxine, and three children: David, Eric, and Judith.

## Conflict of interest statement

The author declares that the research was conducted in the absence of any commercial or financial relationships that could be construed as a potential conflict of interest.
